# Therapy-resistant autoimmune nodopathy with anti-neurofascin 155 antibodies: a case report

**DOI:** 10.3389/fnhum.2024.1405617

**Published:** 2024-07-09

**Authors:** Teodors Talers, Daina Pastare, Guntis Karelis, Eva Sankova

**Affiliations:** ^1^Riga Stradins University, Riga, Latvia; ^2^Department of Neurology and Neurosurgery, Riga East University Hospital, Riga, Latvia

**Keywords:** autoimmune, nodopathy, neurofascin, antibodies, polyneuropathy

## Abstract

This study reports the case of a previously healthy man in his late 20s who began experiencing symptoms 3 months before admission to our hospital, including arm and leg weakness and distal hypesthesia. Initially, the patient responded to corticosteroid therapy. However, as his symptoms progressed, he underwent plasmapheresis and received intravenous immunoglobulin therapy, neither of which led to any discernible improvement. With rapid symptom progression during subsequent hospital visits, further investigation led to the detection of neurofascin 155 antibodies. Based on existing evidence of its efficacy, rituximab treatment was initiated. To date, the patient has received three doses of rituximab, which has been partially ineffective. Thus, treatment is ongoing and includes a combination of rituximab and subcutaneous immunoglobulin.

## 1 Introduction

Chronic inflammatory demyelinating polyneuropathy (CIDP) is an autoimmune neuropathy characterized by symmetrical muscle weakness, both proximal and distal, and sensory loss. It predominantly affects men over women at a ratio of 2:1 and has a prevalence of 2.81 per 100,000 persons. The disease imposes significant economic costs and debilitating effects (Mathey et al., 2015; Broers Merel et al., 2019; Van et al., 2021). Autoimmune antibodies usually target myelin antigens, leading to macrophage activation and resulting in demyelination. However, approximately 20% of patients presenting with CIDP-like symptoms are misdiagnosed. These individuals do not have antibodies against myelin antigens but instead have antibodies targeting paranodal antigens, which affect proteins at the nodes of Ranvier, leading to a slight reduction in myelinated fiber density and the presence of scattered myelin ovoids without the involvement of macrophage-mediated demyelination. In such cases, the myelin sheath is tightly bound to the transverse bands, thus causing autoimmune nodopathy (AN). The most commonly identified antigens in AN include contactin-1, contactin-associated protein-1, and neurofascin-155 (NF-155), with antibodies against NF-155 being the most prevalent among paranodopathies and accounting for 4–18% of CIDP (Dalakas, 2011; King et al., 2012; Koike et al., 2017; Van et al., 2021).

Treatment for CIDP typically involves immunomodulation through intravenous immunoglobulin (IVIg), plasmapheresis (PLEX), and corticosteroids (CS). Additionally, other pharmacological-based immunomodulating therapies, such as rituximab (RTX), have recently been tested in selected cases, particularly in patients with CIDP and AN who are resistant to conventional treatments. Although their efficacy is still unknown, a large-scale randomized trial has yet to be conducted to quantify the usefulness of immunomodulating therapy, so the current body of evidence for efficacy includes only case reports and case report reviews (Hahn et al., 1996; Mendell et al., 2001; Hughes et al., 2008; Benedetti et al., 2010; Eftimov et al., 2013; Nobile-Orazio et al., 2014; Li et al., 2020; Fels et al., 2021). To provide further credible data, we present the case of a man in his late 20s who, after 6 months of conventional treatment-resistant CIDP, was diagnosed with an AN NF-155 subtype, following which he received three doses of RTX as a second-line treatment. Unfortunately, this approach proved ineffective in this case. Treatment was augmented with subcutaneous immunoglobulin (ScIg). His condition was assessed using the Inflammatory Rasch-built Overall Disability Scale (I-RODS), the Inflammatory Neuropathy Cause and Treatment (INCAT) Disability Score, the Overall Neuropathy Limitations Scale (ONLS), and the Medical Research Council (MRC) scale for muscle strength, which evaluates specific muscle groups affected by CIDP and Guillain-Barre syndrome.

## 2 Case description

We present a clinical case of a previously healthy man in his late 20s, with no significant family or medical history, who developed muscle weakness and hypesthesia in his arms and legs approximately 3 months prior to his initial hospital admission. The symptoms progressed, leading to his admission to a regional hospital nearly 1 month before he came to our hospital, where he was treated with high-dose intravenous methylprednisolone (MP) therapy. as his condition improved, he was discharged with a prescription of a daily regimen of 10 mg of prednisolone.

On the day of admission to our hospital (day 0), the patient visited the emergency department and was evaluated by a neurologist. A lumbar puncture was performed, and the collected sample was sent for testing. The test results (Table 1) showed notable total protein and albumin levels, indicating albuminocytologic dissociation, a finding that is consistent with polyneuropathies (Guillain, 1916). However, given the chronic nature of the illness and normal routine blood tests, a more complex condition was considered. Clinically, the patient presented with “glove-sock” type hypesthesia and mild paresis, which was not objectively confirmed (with an MRC sum-score of 30), and he lacked deep tendon reflexes in the legs. Based on these findings, the patient was admitted to the neurology department with a suspected diagnosis of CIDP, and his ongoing prednisolone therapy was discontinued.

**Table 1:** Laboratory test results on day 0 of the first admission.

Cerebrospinal fluid (CSF) analysis	Value	Ref. interval.	Units of measure
Appearance	Colorless, clear	Colorless, clear	-
Total Protein	4,300	250–430	mg/L
Lactate	1.78	1.1–2.4	mmol/L
Glucose	3.69	2.80–4.20	mmol/L
Immunoglobulin G	0.396	0.06–0.08	g/L
Albumin	2971.0	110–350	mg/L
Albumin CSF-serum index	62.29	0.00–6.50	
Cytosis	3	<5	x/μL
Mononuclear leukocytes	3		x/μL
Polymorphonuclear leukocytes	0		x/μL

First, the patient received five rounds of PLEX, but there was no improvement in the patient’s condition. On day 8, with a strong suspicion of CIDP, a 5-day regimen of intravenous immunoglobulin (IVIg) therapy was initiated, administering 30 mg doses daily. Despite interventions, there was no noticeable improvement in the patient’s symptoms by day 14 of admission. Given that responses to treatment, both PLEX and IVIg, can sometimes be delayed, taking anywhere from 10 days to 3 weeks after treatment (Mendell et al., 2001; Hughes et al., 2008; Eftimov et al., 2013), with his clinical presentation remaining unchanged from admission, the patient was discharged in a satisfactory condition (I-RODS 48, INCAT 2, and ONLS 1). He was advised to continue ambulatory treatment under the supervision of his general practitioner and to consult a neurologist after 1 month.

The patient was readmitted to our hospital 52 days after the original presentation, exhibiting significant disease progression. Neurological examination showed dysphonia and normal cranial innervation, was positive for Romberg and Mingazzini tests, indicating an inability to maintain limb symmetry, and showed pronounced muscle weakness in the legs (MRC grade 3 for all muscle groups). Additionally, there was notable atrophy in all extremities, especially in the foot dorsal flexors, tetraparesis, dysmetria evidenced by positive finger-to-nose and heel-to-shin tests, hand tremors, “glove-sock” type hypesthesia, unstable tandem gait, and sensory ataxia in the legs requiring bilateral assistive devices. Deep tendon reflexes in the legs were still absent. Initial assessment scores were 22, 6, and 6 for I-RODS, INCAT, and ONLS. First, the patient was prescribed high-dose methylprednisolone pulse therapy, followed by a daily dose of prednisolone (60 mg). On day 7 of his second hospital admission, corresponding to day 59 of the case, a nerve conduction study was performed. The results showed reduced nerve conduction velocity, absent F-waves, and prolonged motor and sensory distal latencies for all the tested nerves, with motor distal latencies exceeding 50% of the upper limit normal, findings that are consistent with CIDP (Van et al., 2021). On day 15 of his second hospital admission, more blood tests were conducted, which showed that serum protein electrophoresis gamma proteins were below the normal reference range of 7 g/L.

On day 16 of the current admission, MRI scans of the cervical and thoracic regions with intravenous gadolinium contrast showed changes consistent with polyradiculoneuritis. Importantly, blood tests performed at an external laboratory identified the presence of anti-NF-155 immunoglobulin G (IgG) via an immunofluorescence assay, confirming a diagnosis of AN with anti-NF-155. This testing followed guidelines for CIDP cases resistant to conventional treatments, after ruling out other infections, rheumatological, or antibody–antigen-related causes of the patient’s symptoms (Table 2) (Van et al., 2021). On day 19, a CT scan of the thorax and abdomen with intravenous contrast was performed, which did not detect the presence of any oncological processes. On day 22 of the second admission, the possibility of starting off-label use of RTX treatment was evaluated, supported by evidence of its efficacy in treating AN (Rodríguez et al., 2019; Li et al., 2020; Fels et al., 2021) as supported by the guidelines (Van et al., 2021). As there is yet to be defined a specific regimen for the use of RTX in the treatment of AN and CIDP, the approved regimen consisted of two 1,000 mg intravenous doses; the first dose was administered on day 28 of the second admission, that is, day 78 from the original presentation. The second dose was to be administered 2 weeks later with the continuation of oral prednisolone. The starting dose of prednisolone was 60 mg once a day. Subsequently, the dose was lowered by 5 mg every week until dicontinuation. On day 29 of the second admission, day 79 of the clinical case, the patient was discharged with the following improved condition scores: 33 for I-RODS, 3 for INCAT, and 4 for ONLS. Muscle strength assessments showed MRC grades of 3 in the arm proximal and leg distal groups and grades of 4 in the arm distal and leg proximal muscle groups.

**Table 2:** Performed antibody and antigen tests for possible causes of symptoms throughout the clinical case.

	Result
Paranodal antibodies	
Anti-NF-155 IgG IFA	Pos
Anti-NF-140 IgG IFA	Neg
Anti-NF-186 IgG IFA	Neg
Anti-CNTN1 IgG IFA	Neg
Anti-CASPR1 IgG	Neg
Antibody tests for rheumatic causes	
ANA IgG	0.40
Anti-DS-DNA IgM, IgA, and IgG	3.50 U/mL
Anti-cardiolipin IgM, IgA, and IgG (ELISA)	8.20 U/mL
ANCA	Neg
Cryoglobulins	Neg
ENA IgG	0.30
Tissue transglutaminase IgA	0.60 U/mL
Tissue transglutaminase IgG	1.10 U/mL
Anti-titin	Neg
Antibody and antigen tests for infectious causes	
Anti-*Treponema pallidum* IgM/IgG	Neg
HBsAg	Neg
Anti-HBc	Neg
Anti-HCV	Neg
*Mycobacterium tuberculosis* (interferon γ)	Neg
Anti-HIV 1/2	Neg
HIV 1 antigen	Neg
HIV p24 antigen	Neg
VZV DNA (quant.)	Neg
Anti-*Borrelia burgdorferi* IgM	4.4 AU/mL
Anti-*B. burgdorferi* IgG	<5.0 AU/mL
EBV DNA (quant.)	Neg
HSV1/2 DNA (quant.)	Neg
CMV DNA (quant.)	Neg
Enterovirus RNA	Neg
Oncological markers	
CA 15–3	2.48 U/mL
CA 19–9	3.13 U/mL
CA 125	5.07 U/mL
CEA	0.30 ng/mL
HE4	50.80 pmol/L
SCC	1.20 ng/mL
CYFRA 21-1	3.65 ng/mL
PSA	neg
Antiganglioside antibodies	
Anti-MAG IgM IFA	11%
Anti-GM1 IgM/IgG	5%
Anti-GM2 IgM/IgG	23%
Anti-GD1a IgM/IgG	6%
Anti-GD1b IgM/IgG	7%
Anti-GQ1b IgM/IgG	14%
Paraneoplastic neurologic antibodies	
Anti-recoverin	Neg
Anti-amphisine	Neg
Anti-Ri	Neg
Anti-Yo	Neg
Anti-Hu	Neg
Anti-TR	Neg
Anti-GAD65	Neg
Anti-CV2	Neg
Anti-PNMA2	Neg
Anti-SOX1	Neg

NF, neurofascin; IgG, immunoglobulin G; IgM, immunoglobulin M; CNTN, contactin; CASPR, contactin associated protein; ANA, antinuclear antibody; DS, double-stranded; DNA, deoxyribonucleic acid; ANCA, antineutrophilic cytoplasmic antibody; ENA, extractable nuclear antigen; HB, hepatitis B virus; HCV, hepatitis C virus; HIV, human immunodeficiency virus; VZV, varicella-zoster virus; EBV, Epstein barr virus; HSV, human simplex virus; CMV, cytomegalovirus; RNA, ribonucleic acid; CA, cancer antigen; CEA, carcinoembryonic antigen; HE4, human epididymis protein 4; SCC, squamous cell cancer antigen; PSA, prostate-specific antigen; MAG, myelin-associated glycoprotein; GAD, glutamic acid decarboxylase; PNMA, paraneoplastic antigen Ma2; SOX, Sry-like high mobility group box; IFA, indirect fluorescent antibody test.

The patient was readmitted after 2 weeks to receive the second dose of RTX. On readmission, the patient reported a small subjective improvement in his overall condition and strength in the arms and legs. In addition, his symptoms had stabilized without further progression. Neurological examination showed decreased muscle strength (with an MRC sum-score of 19), with more pronounced weakness in the distal than proximal arm muscles (grade 3) and paresis in both legs, notably in the dorsal foot (grade 3). Deep tendon reflexes in the legs were absent. Romberg’s test remained unstable, muscle tone was reduced, and the heel–knee test indicated dysmetria. The patient exhibited polyneuritic-type sensory disturbance, significant deep sensory loss in the feet, sensory ataxia, and an unstable gait. He was able to walk unaided but had a high risk of falling and reported frequent nocturnal urination. His condition scores were 39, 3, and 4 for the I-RODS, INCAT, and ONLS. Three days later, the patient received the second dose of RTX and was discharged to continue care under his general practitioner.

In the interim, before his next consultation, the patient reported a deterioration in his condition. At that point, the patient had been taking 30 mg of prednisone daily for a week; due to his reported decline, his dose was increased to 40 mg to facilitate the continuation of the tapering process.

Approximately 1 month after being discharged from the hospital, the patient was readmitted for the fourth time with complaints of hand tremors, bilateral dysmetria, worsening weakness in his hands and legs, sensory ataxia, periodic shank pain, and increasing instability since his last visit. Muscle strength in the food dorsal flexors had further declined to MRC grade 2. Other distal muscle groups were at grade 3, while proximal muscle groups in all limbs remained at grade 4. His ambulation was reduced to just a few meters, even with bilateral support, scoring 23 on I-RODS, 5 on INCAT, and 5 on ONLS. The following day, a nerve conduction study was performed, which showed motor and sensory axonal neuropathy alongside marked demyelinating polyneuropathy, which was particularly severe in the legs compared to the arms. This indicated a significant progression from previous assessments. In response, the patient was prescribed 1,000 mg of intravenous methylprednisolone daily for 5 days. After the treatment, the patient reported a subjective feeling of improvement; however, objectively, the improvement was negligible.

Due to the patient’s weakening state, a choice was made to start treatment with subcutaneous immunoglobulin. This duration of his fourth hospital admission lasted a total of 2 weeks, and in accordance with the patient’s wishes, he was discharged. At the time of discharge, his neurological assessment showed severe limitations: he could hardly move, even with the aid of a high table, and exhibited tetraparesis, which was more pronounced distally (MRC grade 2) than proximally (grade 4). He tested positive for Romberg’s test, displayed severe sensory ataxia in all limbs, and displayed deep sensory loss in the feet. Deep tendon reflexes in the legs were absent. His condition scores were 18 for I-RODS, 5 for INCAT, and 6 for ONLS.

On day 181 of the case, the patient consulted with his neurologist. He was evaluated for the possibility of starting subcutaneous immunoglobulin injections. Moreover, changes were made to his prednisone therapy: for a week, the dosage was reduced from 45 mg to 40 g, and after that, the dose was to remain unchanged until further review.

On day 205 of the case, the patient was admitted for a day to start subcutaneous immunoglobulin therapy. His neurological state had improved since the previous admission. At this time, he required only unilateral support for walking, with muscle strength graded as MRC 4 proximally and MRC 3 distally in all limbs. However, Romberg’s test remained positive, and deep tendon reflexes were still absent in both legs. He received his first 10 mg dose of subcutaneous immunoglobulin without any adverse effects. He received training on the self-administration of ScIg and was discharged with improved condition scores of 28 for the I-RODS, 3 for INCAT, and 5 for ONLS.

The latest admission on day 288 showed a marked improvement in the patient’s condition. The most significant progress was observed in muscle strength; all muscle groups reached an MRC grade of 5, except for the foot dorsal flexors, which remained at grade 4. Despite these improvements, Romberg’s test was still positive, and deep tendon reflexes were still absent in both legs. The patient also experienced polyneuritic-type paraesthesia, disturbances in proprioception, and impaired vibration sensation, indicating persistent sensory ataxia. On the second day of the latest admission, he received the third dose of RTX. On day 3, he was once again discharged.

## 3 Timeline

See Figures 1, 2.

**Figure 1:**
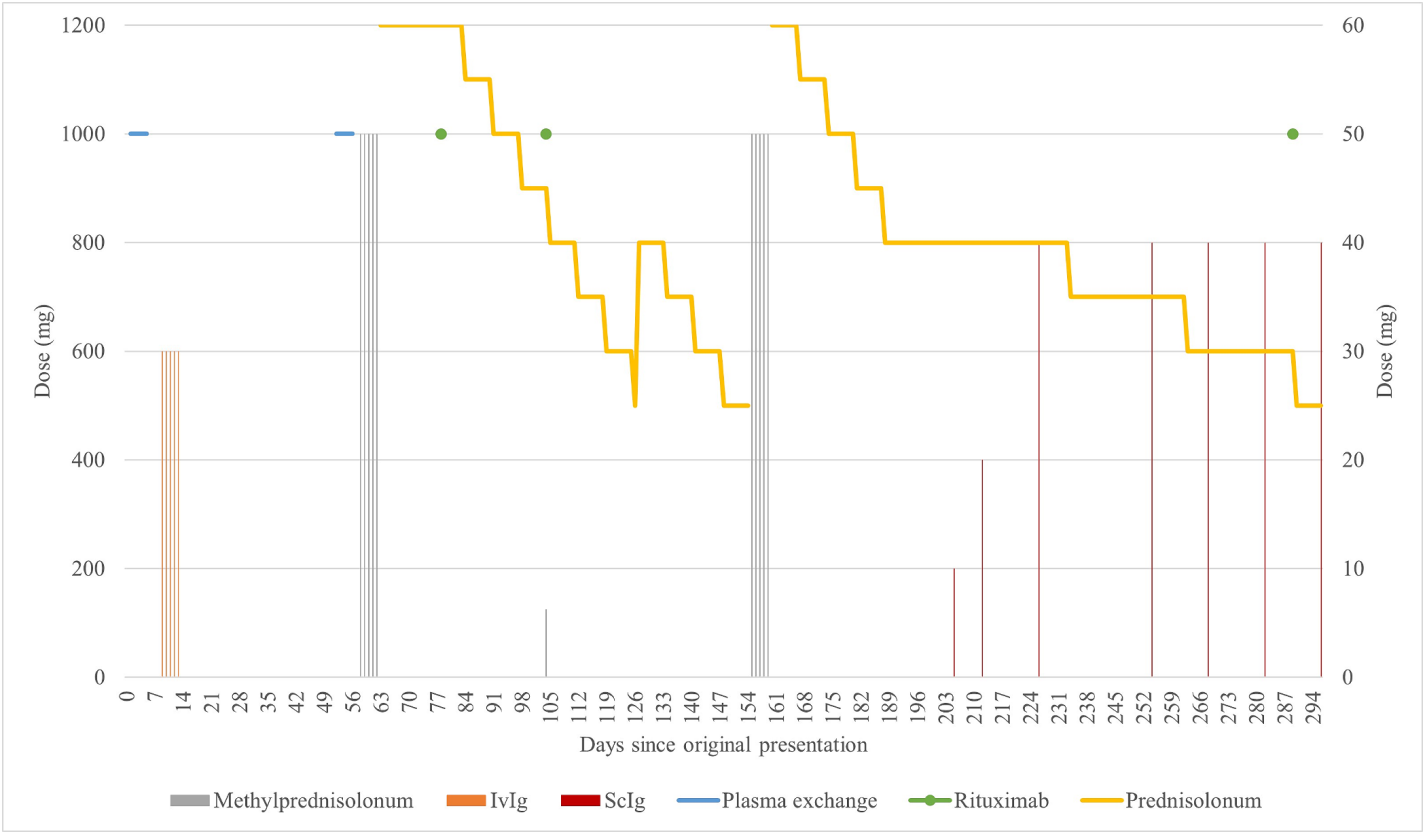
Graphical summarization of the treatment. The scale of doses for methylprednisolone (grey) and rituximab is shown on the left side of the graph. The scale of doses for prednisone (yellow), IVIg (orange), and ScIg (dark red) is shown on the right side of the graph. Plasma exchange (light blue) is shown for illustrative purposes as to when it was performed.

**Figure 2:**
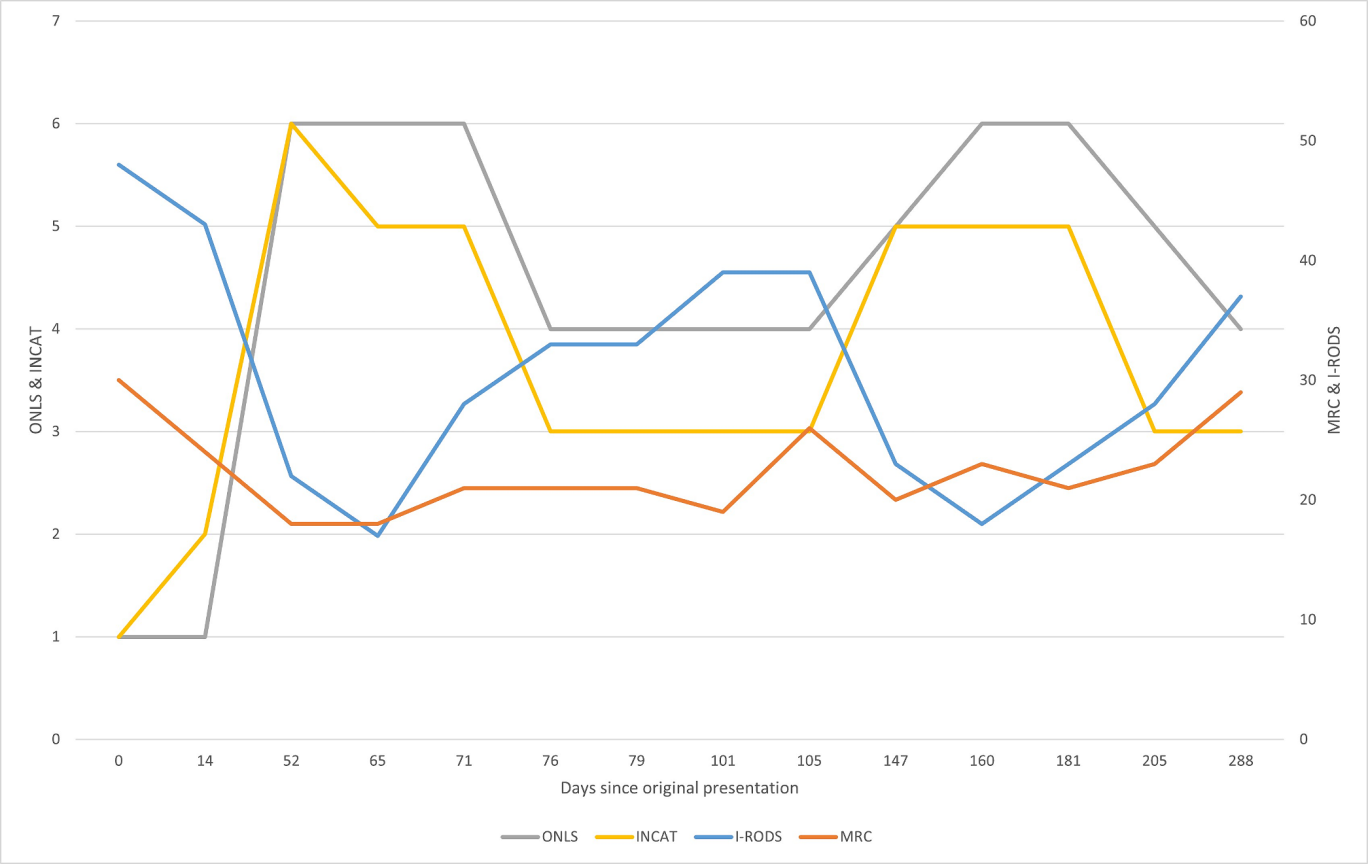
Graphical summarization of the used evaluation scales. The sum scores for the scales ONLS (grey) and INCAT (yellow) are shown on the left side of the graph. The sum scores for the scales I-RODS (light blue) and MRC (orange) are shown on the right side of the graph. For the MRC scale, the more sensitive muscle groups were picked to be evaluated and comprise the shown sum: upper arm abductors, elbow flexors, wrist extensors, hip flexors, knee extensors, and foot dorsal flexors (Kleyweg et al., n.d.). The figure represents changes in evaluation scores throughout the clinical case. By comparing with Figure 1, primary improvement was observed following corticosteroid therapy, and the following declines can be attributed to prednisone dose reduction, from 30 mg/day and lower. There seemed to be an improvement after rituximab treatment in the first 24 days (between days 79 and 103), but following that, there was a decline in the patient’s state as the prednisone dose was reduced. The improvement after starting rituximab should typically be seen after 2 months of treatment, yet there was no clear improvement. Substantial improvement became apparent after starting treatment with subcutaneous immunoglobulin, which is apparent in the evaluation scores and reduction in the prednisone dose following day 205.

## 4 Diagnostic assessment

See Tables 1, 2.

## 5 Discussion

CIDP is an autoimmune disease characterized by progressive or relapsing symmetric muscle weakness that affects both proximal and distal muscles of the upper and lower extremities, sensory impairment in at least two extremities, which develop over 8 weeks, and deep tendon reflexes that are absent or reduced. Atypical clinical forms of this disease may present with isolated sensory or motor symptoms or manifest focally, multifocally, or distally (Van et al., 2021). AN, while sharing many characteristics of CIDP, differs primarily in the targets of its associated antibodies. Unlike CIDP, where antibodies target myelin antigens, AN involves antibodies against paranodal antigens, which affect proteins at the nodes Ranvier, where the myelin sheath is tightly bound to the transverse bands, leading to similar symptoms. Clinically, AN tends to be more motor-dominant than sensory, affects more distal than proximal regions, and is often accompanied by sensory ataxia and tremor (Dalakas, 2011; King et al., 2012; Rodríguez et al., 2019; Van et al., 2021).

Current diagnostic guidelines for CIDP are based on clinical and electrodiagnostic criteria. Clinically, the patient’s symptoms must align with the typical or atypical variants of the disease. Electrodiagnostically, abnormalities must be present in at least two motor nerves that meet the motor conduction criteria, and sensory conduction abnormalities must also be present in at least two nerves (Van et al., 2021). In our case, based on the aforementioned criteria, the diagnosis of CIDP was irrefutable as the patient’s symptoms had been progressing over a period of 10–12 weeks, since before his first hospital admission, and, on first admission, all tendon reflexes were absent. Additionally, the nerve conduction study for motor tests showed that distal latency in all six major nerves exceeded 50% of the upper limit of normal, meeting the electrodiagnostic criteria. According to EAN/PNS guidelines, the CIDP diagnosis in this clinical case was confirmed. The diagnosis of AN is more challenging than CIDP because, clinically, AN has a striking resemblance to CIDP, and the confirmation of antibody presence is required, which is not commonly conducted (Van et al., 2021). In the early stages of the case, a lumbar puncture showed a substantial increase in total protein and albumin levels and no cytosis. The albumin and cell count changes point to albuminocytological dissociation, which is more specific to CIDP but also prevalent in AN cases. Notably, the rise in total protein quantity is more pronounced in AN, with the median level, on average, reported as nearly three times higher than in CIDP cases (Guillain, 1916; Ogata et al., 2015).

CIDP is an autoimmune complex disease with no specific etiotropic treatment options. The empirical treatment options include IVIg, PLEX, CS, and, a relatively new addition, monoclonal antibody medications. IVIg therapy was shown to be effective in 44–63% of cases (Mendell et al., 2001; Hughes et al., 2008; Eftimov et al., 2013), while PLEX shows effectiveness in approximately 80% of cases (Hahn et al., 1996). IVIg also has a higher primary response rate than CS, with effectiveness ranging from 87.5 to 54.2% (Nobile-Orazio et al., 2014). Recent advancements indicate that ScIg is a safer alternative to IVIg treatment for maintenance in patients who respond to IVIg. ScIg offers higher patient satisfaction and fewer, less severe side effects compared to IVIg. It has a time to response (TTR) of 4 weeks, provided it is started within 1 week of the last IVIg dose (Van et al., 2018; Goyal et al., 2021). Although head-to-head trials have not yet been conducted to directly compare both forms of immunoglobulin therapies, current data suggest that their efficacy is comparable (Goyal et al., 2021).

However, treatment strategies differ for AN. For example, cases involving anti-NF-155 and anti-contactin 1 antibodies show some resistance to IVIg therapy. These antibodies primarily belong to the IgG4 subclass of immunoglobulins, which is known for their low capacity to bind Fc receptors and low affinity for activating complements. This characteristic could explain the observed resistance to IVIg and ScIg, as the efficacy of ScIg depends on the initial efficacy of IVIg and is typically used as a maintenance treatment alternative to IVIg (Van et al., 2018; Bunschoten et al., 2019; Lehmann et al., 2019). It has also been shown that PLEX should be avoided in cases of anti-NF 155 and anti-contactin as it is ineffective in removing IgG4 from plasma (Kuwahara et al., 2017; Koike and Katsuno, 2020).

The aforementioned points show the need to detect the antibodies causing symptoms, as it does change the treatment approach. In this case, CS therapy appeared to be the only treatment to which the patient responded, supporting the data on resistance to classical treatment options for CIDP, but as the disability continued to progress, other treatment options were sought. Therefore, the choice was made to begin RTX therapy. Although, at the time of writing this report, large-scale randomized trials evaluating the efficacy of RTX in treating CIDP and AN have not yet been performed, evidence suggests that RTX can be effective, especially in cases where patients do not respond to conventional treatment (Benedetti et al., 2010) and in those with anti-NF-155 antibodies (Li et al., 2020; Fels et al., 2021).

After beginning RTX treatment, the patients showed improvement during the first 24 days (between days 79 and 103), as assessed through subjective and objective evaluations. However, on day 148, there was a substantial decline in evaluation scores. The reported time to TTR for RTX treatment plans was 1–3 months, with improvements lasting up to 1 year and peaking between 2 and 18 months (Benedetti et al., 2010; Muley et al., 2020). Unfortunately, the patient’s condition worsened roughly 2 months after starting rituximab, which does not coincide with the available data on TTR, but the worsening of the conditions seems to correlate with the dose of prednisone, especially when the dose fell below 30 mg/day.

The typical TTR for prednisolone therapy is 4–8 weeks (Barohn et al., 1989; Van et al., 2021), when this for of therapy is to be evaluated. Dosage adjustments are also important. It has been noted that patients who started with a dose of 40 mg/day often showed improvement only after increasing their dose to the range of 60–110 mg/day (Wertman et al., 1988), which seems to provide a therapeutic dose range. In the same study, Wertman et al. (1988) reported that patients who started on steroid therapy (1.0–1.5 mg/kg) and later tapered off experienced a clinical relapse within a median of 8.4 weeks after lowering the dose. The decline in the condition of our patient seems to support these data.

The beginning of RTX treatment coincided with the beginning of the prednisone tapering process, and 2 months later, i.e., roughly 8 weeks, the decline. Due to the decline in the patient’s condition, we opted for a trial use of ScIg, recognizing that a single treatment course may not accurately reflect the overall effectiveness of the treatment option despite the reported data on the mechanism of resistance. Currently, the patient’s condition is improving; muscle strength in all muscle groups has remained at no lower than grade 4. This is the first instance since the initial presentation where ScIg and RTX were administered concurrently, with ongoing prednisone treatment reduced to 25 mg/day, and no further declines have been observed.

Based on the currently available data, anti-NF-155 poses a great challenge for patients as well as physicians. Only a timely diagnosis and the delivery of proper treatment can provide a better outcome, as routine treatments seem to be ineffective, except for CS. Further research should explore the pharmacological basis for why RTX treatment is effective in some cases and not in others.

## 6 Takeaway messages

- In the event of a CIDP diagnosis and treatment resistance, further immunological investigation is warranted.
- Patients with positive NF antibodies are not classified as having CIDP.
- Autoimmune nodopathies are resistant to conventional therapy for CIDP.
- Even second-line treatment with RTX may be insufficient and may need be combined with ScIg.

## 7 Patient perspective

Looking back, I realize that the first symptoms appeared as early as the beginning of the year when I was plagued by constant fatigue. At first, I thought it was due to the increased workload and that it would pass. Fatigue did not go away, and then I developed pain in my leg muscles, which was similar to pain after a difficult workout. I wanted to stretch all the time but felt no relief. When one of my legs started to slightly tingle a few weeks later, I made an appointment with my general practitioner, who referred me to a neurologist and ran some blood tests. Soon after that, both feet began tingling, and I started to worry that it might be something serious. Two months later, the neurologist referred me for an electrical examination of my nerves, which showed that they were damaged, and the next day, I was admitted to the nearest hospital. I was frightened and slept poorly at night. The most frightening thoughts were about the uncertainty about my future, my wife, and my little boy. I spent a week in the hospital, and after my condition improved rapidly, I thought I would soon be able to go back to work and resume hockey training. The treating physician assured me that I had Guillain-Barre syndrome and that everything would improve now. I spent a few days at home, but my symptoms worsened rapidly. I thought I was going to lose my mind. I visited several neurologists, but they advised me to continue with the treatment and assured me that there was nothing to worry about. I also tried to make an appointment at the Rare Diseases Center, but the waiting list was 6 months long. All of it was hard to digest. Although the doctor who treated me told me everything was clear and that I just needed to get well, my condition continued to worsen. I am grateful to the doctor who consulted me outside of her office hours and recommended that I go to another hospital. The time I spent in the largest hospital in my country was very difficult, both emotionally and physically. I was away from my family, and I continued to become weaker. In the hospital, I was treated with plasma exchange and immunoglobulins. I left the hospital in a terrible condition. I could barely walk 100 m. I cried a lot in the evenings and could not sleep. I started to use crutches to move around and took a lot of drugs, but it only became worse. The doctors probably already guessed my diagnosis but did not tell me. I did not know what the plan was, and I was very scared.

When I returned to the hospital 2 months later, I was barely able to move on crutches and was almost only able to be in a supine position. I did not know if I would ever be able to play ball with my son again or be a useful member of society in the future. I cried a lot. I was happy when I was transferred to another ward. The atmosphere was homier, and the attitude was more personal. I received steroids, and my condition improved rapidly, including a clear diagnosis of AN with an NF155 antibody. The world seemed rosy, the sun was shining, and the birds were chirping. I also received my first dose of Rituximab, and everything seemed very good. The joy lasted only a few weeks; the symptoms returned, and the depression was back. My only hope was that Rituximab would take effect later. I read on forums that sometimes it takes as long as 3 months. When I was hospitalized the next time, it seemed that every day would be this low point, and all I had to do was move upward, and I believe that happened. Once again, steroids were administered, but the expected rapid improvement did not follow. Since then, I think my abilities have slowly improved without marked jumps until today. I have been back at work for over a month now and have not taken any painkillers since last year. I exercise a lot, and I hope to be able to play hockey again. I still need to work on my balance and overall strength, and my fingers are not fully functional, but they are not that important. Now, I am also sorting out my disability. To sum up, my wife and my little boy helped me find the strength to fight and not give up. I dreaded thinking about what would happen to me if I did not have them. All in all, I feel very well now, and I am eternally grateful for what has been given.

## Data availability statement

The original contributions presented in the study are included in the article/Supplementary material. Further inquiries can be directed to the corresponding author/s.

## Ethics statement

The studies involving humans were approved by the Rīga Stradiņš University Research Ethics Committee. The studies were conducted in accordance with local legislation and institutional requirements. The participants provided their written informed consent to participate in this study. Written informed consent was obtained from the individual(s) for the publication of any potentially identifiable images or data included in this article.

## Author contributions

TT: Visualization, Writing – original draft, Writing – review & editing. DP: Supervision, Writing – original draft, Writing – review & editing. GK: Supervision, Writing – original draft, Writing – review & editing. ES: Writing – original draft, Writing – review & editing.

## References

[ref1] Barohn R. J. Kissel J. T. Warmolts J. R. Mendell J. R. (1989). Chronic inflammatory demyelinating Polyradiculoneuropathy. Arch. Neurol. 46, 878–888. doi: 10.1001/archneur.1989.00520440064022 2757528

[ref2] Benedetti L. Briani C. Franciotta D. Fazio R. Paolasso I. Comi C.. (2010). Rituximab in patients with chronic inflammatory demyelinating polyradiculoneuropathy: a report of 13 cases and review of the literature. J. Neurol. Neurosurg. Psychiatr. 82, 306–308. doi: 10.1136/jnnp.2009.188912 20639381

[ref3] Broers Merel C. Bunschoten C. Nieboer D. Lingsma Hester F. Jacobs B. C. (2019). Incidence and prevalence of chronic inflammatory demyelinating Polyradiculoneuropathy: a systematic review and Meta-analysis. Neuroepidemiology 52, 161–172. doi: 10.1159/000494291, PMID: 30669140 PMC6518865

[ref4] Bunschoten C. Jacobs B. C. Van P. Cornblath D. R. Doorn van P. A. (2019). Progress in diagnosis and treatment of chronic inflammatory demyelinating polyradiculoneuropathy. Lancet Neurol. 18, 784–794. doi: 10.1016/S1474-4422(19)30144-9 31076244

[ref5] Dalakas M. C. (2011). Advances in the diagnosis, pathogenesis and treatment of CIDP. Nat. Rev. Neurol. 7, 507–517. doi: 10.1038/nrneurol.2011.121 21844897

[ref6] Eftimov F. Winer J. Vermeulen M. De Haan R. Van S. (2013). Intravenous immunoglobulin for chronic inflammatory demyelinating polyradiculoneuropathy. Cochrane Library. doi: 10.1002/14651858.CD001797.pub3/epdf/full 24379104

[ref7] Fels M. Fisse A. L. Schwake C. Motte J. Athanasopoulos D. Grüter T.. (2021). Report of a fulminant anti-pan-neurofascin-associated neuropathy responsive to rituximab and bortezomib. J. Peripheral Nerv. Syst. 26, 475–480. doi: 10.1111/jns.12465 34486194

[ref8] Goyal N. Karam C. Sheikh K. A. Dimachkie M. M. (2021). Subcutaneous immunoglobulin treatment for chronic inflammatory demyelinating polyneuropathy. Muscle Nerve 64, 243–254. doi: 10.1002/mus.27356, PMID: 34260074 PMC8457117

[ref9] Guillain G. (1916). Sur un syndrome de radiculonevrite avec hyperalbuminose due liquide cephalo-rachidien sans reaction cellulaire. Remarque sur les caracteres clinique et graphiques des reflezes tendinuex. Bull et mem Soc med d hop de Paris 40:1462. 10400560

[ref10] Hahn A. F. Bolton C. F. Pillay N. Chalk C. Benstead T. Bril V.. (1996). Plasma-exchange therapy in chronic inflammatory demyelinating polyneuropathy: a double-blind, sham-controlled, cross-over study. Brain 119, 1055–1066. doi: 10.1093/brain/119.4.1055, PMID: 8813270

[ref11] Hughes R. Donofrio P. D. Bril V. Dalakas M. C. Deng C. Kim H.. (2008). Intravenous immune globulin (10% caprylate-chromatography purified) for the treatment of chronic inflammatory demyelinating polyradiculoneuropathy (ICE study): a randomised placebo-controlled trial. Lancet Neurol. 7, 136–144. doi: 10.1016/S1474-4422(07)70329-0, PMID: 18178525

[ref12] King J. Malotka J. Kawakami N. Derfuss T. Khademi M. Olsson T.. (2012). Neurofascin as a target for autoantibodies in peripheral neuropathies. Neurol. Int. 79, 2241–2248. doi: 10.1212/WNL.0b013e31827689ad PMC3542349 23100406

[ref13] Kleyweg R. P. Der V. Schmitz M. Interobserver agreement in the assessment of muscle strength and functional abilities in Guillain-Barré syndrome. PubMed 14, 1103–1109. doi: 10.1002/mus.880141111 1745285

[ref14] Koike H. Kadoya M. Kaida K. I. Ikeda S. Kawagashira Y. Iijima M.. (2017). Paranodal dissection in chronic inflammatory demyelinating polyneuropathy with anti-neurofascin-155 and anti-contactin-1 antibodies. J. Neurol. Neurosur. Psychiatr. 88, 465–473. doi: 10.1136/jnnp-2016-314895 28073817

[ref15] Koike H. Katsuno M. (2020). Pathophysiology of chronic inflammatory demyelinating polyneuropathy: insights into classification and therapeutic strategy. Neurol. Ther. 9, 213–227. doi: 10.1007/s40120-020-00190-8#ref-CR11, PMID: 32410146 PMC7606443

[ref16] Kuwahara M. Suzuki H. Oka N. Ogata H. Yanagimoto S. Sadakane S.. (2017). ELectron microscopic abnormality and therapeutic efficacy in chronic inflammatory demyelinating polyneuropathy with anti-neurofascin155 immunoglobulin G4 antibody. Muscle Nerve 57, 498–502. doi: 10.1002/mus.25757 28796305

[ref17] Lehmann H. C. Burke D. Kuwabara S. (2019). Chronic inflammatory demyelinating polyneuropathy: update on diagnosis, immunopathogenesis and treatment. J. Neurol. Neurosurg. Psychiatr. 90, 981–987. doi: 10.1136/jnnp-2019-320314 30992333

[ref18] Li J. Xiang Y. Li S. Zhang F. Ruan X. Guo S. (2020). Efficacy of low dose rituximab in treatment-resistant CIDP with antibodies against NF-155. J. Neuroimmunol. 345:577280. doi: 10.1016/j.jneuroim.2020.577280 32563125

[ref19] Mathey E. K. Park S. B. Hughes R. A. Pollard J. D. Armati P. J. Barnett M.. (2015). Chronic inflammatory demyelinating polyradiculoneuropathy: from pathology to phenotype. J. Neurol. Neurosurg. Psychiatr. 86, 973–985. doi: 10.1136/jnnp-2014-309697 PMC4552934 25677463

[ref20] Mendell J. R. Barohn R. J. Freimer M. Kissel J. T. King W. Nagaraja H. N.. (2001). Randomized controlled trial of IVIg in untreated chronic inflammatory demyelinating polyradiculoneuropathy. Neurol. Int. 56, 445–449. doi: 10.1212/WNL.56.4.445 11222785

[ref21] Muley S. Jacobsen B. Parry G. Usman U. Ortega E. Walk D.. (2020). Rituximab in refractory chronic inflammatory demyelinating polyneuropathy. Muscle Nerve 61, 575–579. doi: 10.1002/mus.26804, PMID: 31922613

[ref22] Nobile-Orazio E. Cocito D. Jann S. Uncini A. Messina P. Antonini G.. (2014). Frequency and time to relapse after discontinuing 6-month therapy with IVIg or pulsed methylprednisolone in CIDP. J. Neurol. Neurosurg. Psychiatr. 86, 729–734. doi: 10.1136/jnnp-2013-307515 25246645

[ref23] Ogata H. Yamasaki R. Hiwatashi A. Oka N. Kawamura N. Matsuse D.. (2015). Characterization of IgG4 anti-neurofascin 155 antibody-positive polyneuropathy. Annals Clinical Transl. Neurol. 2, 960–971. doi: 10.1002/acn3.248, PMID: 26478896 PMC4603379

[ref24] Rodríguez Y. Vatti N. Ramírez-Santana C. Chang C. Óscar Mancera-Páez M. Gershwin E.. (2019). Chronic inflammatory demyelinating polyneuropathy as an autoimmune disease. J. Autoimmunity 102, 8–37. doi: 10.1016/j.jaut.2019.04.021 31072742

[ref25] Van S. Bril V. van Geloven N. Hartung H. Lewis R. A. Sobue G.. (2018). Subcutaneous immunoglobulin for maintenance treatment in chronic inflammatory demyelinating polyneuropathy (PATH): a randomised, double-blind, placebo-controlled, phase 3 trial. Lancet Neurol. 17, 35–46. doi: 10.1111/jns.12455 29122523

[ref26] Van P. Van D. RDM H. Avau B. Vankrunkelsven P. Allen J. A.. (2021). European academy of neurology/peripheral nerve society guideline on diagnosis and treatment of chronic inflammatory demyelinating polyradiculoneuropathy: report of a joint task force—second revision. J. Peripheral Nerv. Syst. 26, 242–268. doi: 10.1111/jns.12455 34085743

[ref27] Wertman E. Argov Z. Abrmasky O. (1988). Chronic inflammatory demyelinating Polyradiculoneuropathy: features and prognostic factors with corticosteroid therapy. Eur. Neurol. 28, 199–204. doi: 10.1159/000116266 3416887

